# Hydrocortisone malabsorption due to polyethylene glycols (Macrogol 3350) in a girl with congenital adrenal insufficiency

**DOI:** 10.1186/s13052-014-0078-2

**Published:** 2014-09-26

**Authors:** Stefano Stagi, Paolo Del Greco, Franco Ricci, Chiara Iurato, Giovanni Poggi, Salvatore Seminara, Maurizio de Martino

**Affiliations:** Department of Health Sciences, University of Florence, Anna Meyer Children’s University Hospital, Florence, Italy

**Keywords:** Macrogol, Hydrocortisone, Adrenal insufficiency, Malabsorption, Polyethylene glycol, Constipation

## Abstract

**Background:**

Primary adrenal insufficiency is relatively rare in children and, if unrecognized, may present with cardiovascular collapse, making it a potentially life-threatening entity.

**Case presentation:**

The proposita, 11 months old of age, was admitted for lethargy and severe dehydration. Blood pressure was 62/38 mm Hg, and biochemical measurements showed hyponatraemia, hypochloraemia, hyperkalaemia, and metabolic acidaemia. Renin activity was 1484 μU/mL; cortisol, 1.03 μg/dL (normal, 5-25 μg/dL); and corticotropin (ACTH), 4832 ng/L (normal, 9-52 ng/L). Adrenal deficiency was diagnosed, and replacement therapy with glucocorticoids and mineralocorticoids was initiated. After 40 days, ACTH was 797 ng/L.

During follow-up, the patient started taking macrogol twice daily for constipation and experienced a significant increase in ACTH (3262 ng/L), which dropped to 648 ng/L when macrogol was stopped. After arbitrary reintroduction of macrogol, the child presented with hypoglycaemia, lethargy, weakness, and hypotonia; ACTH was 3145 ng/L. After again stopping macrogol, her ACTH was near normalized (323 ng/L).

**Conclusion:**

Hydrocortisone malabsorption may be caused by macrogol use. Because chronic constipation is frequently reported in children, the possibility that macrogol contributes to adrenal crisis should be taken in account.

## Introduction

Adrenal insufficiency is relatively rare in children and may be categorized as primary or secondary and congenital or acquired [1]. Primary adrenal insufficiency can be caused by a deficiency in steroid biosynthesis or abnormal adrenal gland development. It is a life-threatening disorder that can result from primary adrenal failure or secondary adrenal disease resulting in impairment of the hypothalamic-pituitary axis. Prompt diagnosis and urgent mineralocorticoid and glucocorticoid replacement is mandatory [2]; however, correct management is also essential [3].

Chronic idiopathic constipation is frequently reported and reduces patient quality of life [4,5]. In fact, chronic constipation is associated with long-term problems including megarectum, reduced sensitivity of the rectum to the presence of faeces, and abnormal gut motility [4]. In many children, constipation is triggered by painful bowel movements caused by factors such as toilet training, changes in routine or diet, stressful events, intercurrent illness, or delaying defecation [4]. Therefore, managing chronic constipation in children effectively and early in its course is important in preventing long-term defecation disorders [4].

Polyethylene glycols (PEGs, or macrogols) are hydrophilic polymers of ethylene oxide [6] used in many drugs such as bowel preparations, dispersing agents, and excipients, and in cosmetics [7]. Water makes up 75-80% (wt/wt) of the normal stool, and a difference of only 10% in hydration results in marked changes in stool consistency [8]. Because PEG is a large molecular weight water-soluble polymer, it has the capacity to form hydrogen bonds with 100 molecules of water per molecule of PEG [9]. When PEG is administered orally, the resulting hydration of the colonic content facilitates transit and painless defecation in a linear dose-dependent fashion [10]. Therefore, PEG-based laxatives, when used in escalating doses, can also be used to completely remove faecal loading in preference to rectally-administered treatments. Standard management of chronic constipation tends to begin with correction of dietary and lifestyle factors that predispose to the condition and focus on increasing dietary fibre and fluid intake [11]. Dietary manipulation alone, including the use of corn syrup, was successful in resolving all symptoms of constipation in 25% of children aged up to 2 years in one US study [5].

We describe a girl with adrenal insufficiency managed with hydrocortisone and fluorocortisone who showed an adrenal crisis after administration of macrogol 3350, and we discuss this aspect, focusing on the aetiology of adrenal insufficiency in childhood.

## Case report

The proposita, 11 months old of age, was admitted to Anna Meyer Children’s University Hospital for lethargy and severe dehydration without history of vomiting or diarrhoea. She was the first child of non-consanguineous, young, healthy Italian parents, born at term (39 wks of gestation) by natural childbirth. Birth weight was 3200 g (0.12 standard deviation score [SDS], 50^th^-75^th^ centile), length, 51 cm (1.12 SDS, 75^th^-90^th^ centile), and head circumference, 35 cm (1.04 SDS, 75^th^-90^th^ centile). There were no perinatal problems or familial history of similar presentations or features of endocrine disease. Neuromotor development was normal; she was sitting at 5 months.

At 10 months, 20 days of age, she started showing weight loss, lethargy, weakness, hypotonia, and dark skin. She was mildly dehydrated. Her body weight, length, and head circumference were 10.850 kg (1.89 SDS, 97^th^ centile), 73 cm (0.61 SDS, 50^th^-75^th^ centile), and 46.5 cm (1.30 SDS, 90^th^ centile), respectively. There were no dysmorphic features. External genitalia were normal female type with no ambiguity. There was no abdominal or inguinal mass discovered upon abdominal examination. Blood pressure was 62/38 mm Hg; respiration, 35/min; pulse, 121/min; and body temperature, 37.3°C.

**Figure 1:**
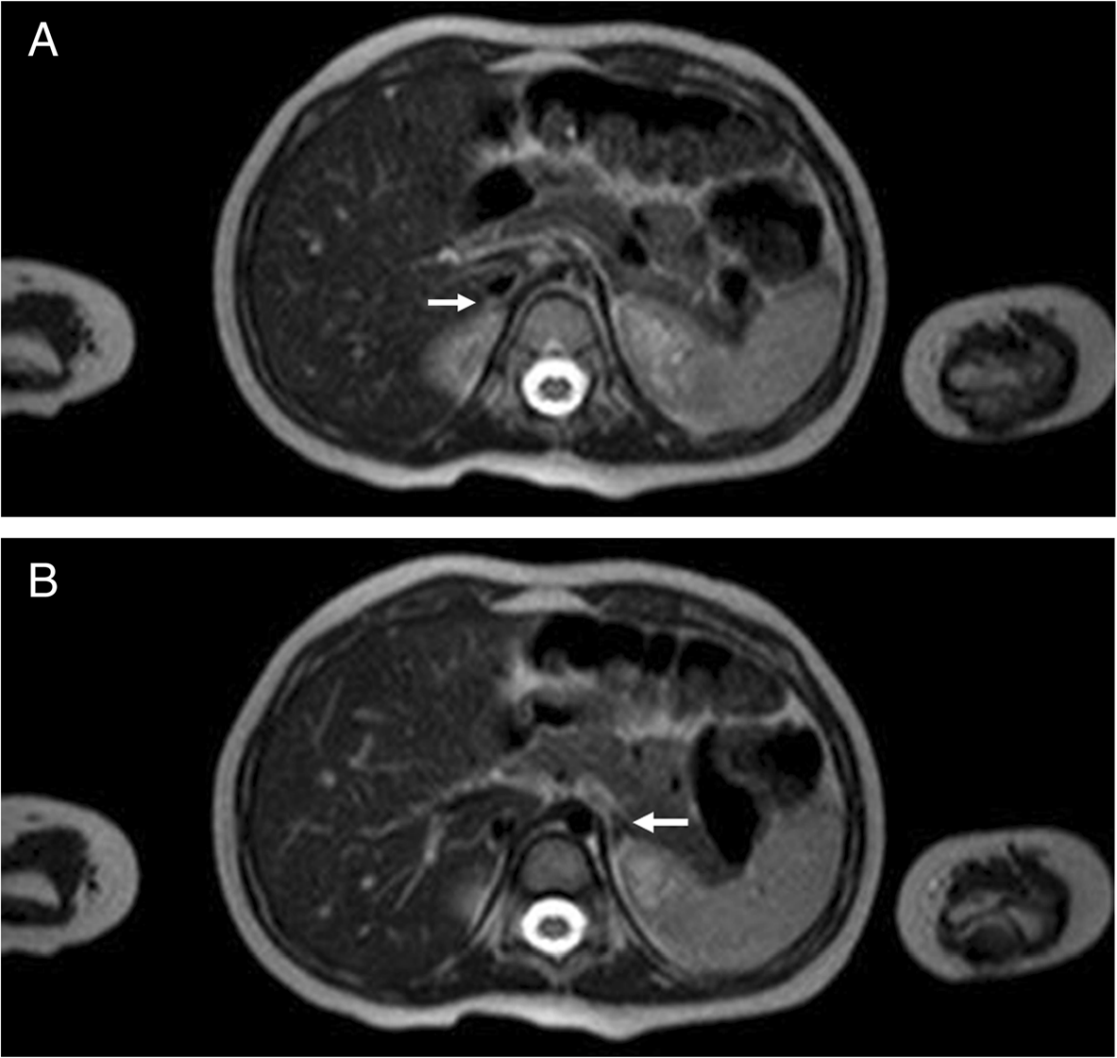
**MRI scan of the bilateral adrenal glands revealing agenesis of the right (A) and hypoplasia of the left (B) adrenal glands.**

Biochemical measurements indicated hyponatraemia (Na, 125 mEq/L), hypochloraemia (Cl, 86 mEq/L), hyperkalaemia (K, 5.7 mEq/L), metabolic acidaemia by arterial venous blood gas, elevated serum urea nitrogen (60 mg/dL), and normal creatinine (0.3 mg/dL). Glucose was 56 mg/dL (normal, 55-110 mg/dL). An extensive endocrine work-up, carried out at 8 am after an overnight fast, showed free thyroxin was 1.27 ng/dL (normal, 0.80-1.90 ng/dL); thyrotropin, 3.96 μIU/dL (normal, 0.4-4.0 μIU/dL); aldosterone, 0.19 nmol/L (normal, 0.96-8.31 nmol/L); renin activity, 1484 μU/mL per h (normal, 2-10.2 μU/mL per h); 17-OH-progesterone, < 0.5 nmol/L; dehydroepiandrosterone sulfate, < 15 μg/dL; cortisol, 1.03 μg/dL (normal, 5-25 μg/dL); ACTH, 4832 ng/L (normal, 9-52 ng/L; Figure 1), luteinizing hormone, 2.3 IU/L; and follicle stimulating hormone, 5.6 IU/L.

Serum cortisol and plasma ACTH levels were measured routinely using an Immulite 2000 chemiluminescence immunometric assay (Diagnostic Products Corporation, xLos Angeles, CA, USA). The cortisol inter-assay and intra-assay coefficients of variation were < 9.5% and 7.4%, respectively. The ACTH inter-assay and intra-assay coefficients of variation ranged from 6.1% to 10.0% and from 6.7% to 9.5%, respectively.

The patient was hydrated with normal saline and required vasopressors. Adrenal deficiency was diagnosed considering the hyponatraemia, hyperkalaemia, metabolic acidaemia, and cortisol and corticotropin levels.

The usual causes of primary adrenal insufficiency were ruled out (Table 1). Family history was negative for autoimmune diseases and endocrinological or genetic syndromes. Renal Doppler ultrasonography was performed and was normal. Autoimmune Addison, in the context of autoimmune polyendocrinopathy candidiasis ectodermal dystrophy (APECED) or other autoimmune syndromes was ruled out by clinical and biochemical evaluation. Mantoux was negative and Veneral Disease Research Laboratory (VDRL) was non-reactive. Human immunodeficiency virus was seronegative. Plasma levels of very long chain fatty acids (VLCFAs) were normal. We performed a synthetic ACTH stimulation test intravenously at 8 am after an overnight fast, and the cortisol response, measured at 0, 30, and 60 minutes after infusion was blunted (0.98, 1.39, and 2.01 μg/dL, respectively). An MRI scan of the bilateral adrenal glands revealed agenesis of the right and hypoplasia of the left adrenal glands (Figures 1A and B).

**Table 1:** **Typical causes of primary adrenal insufficiency**

**1) Genetic disorders**	**OMIM** ^**1**^ **(gene map)**	**Etiologic mechanisms**	**Other signs and symptoms**
Adrenoleukodystrophy	300100 (Xq28)	Mutations of *ABCD1* ^2^, *ABCD2* ^3^	Weakness, diminished visual acuity, deafness, cerebellar ataxia, hemiplegia, convulsions, dementia
Congenital adrenal hyperplasia			
*21*-hydroxylase deficiency	201910 (6p21.33)	Mutations of *CYP21A2* ^4^	Hyperandrogenism, ambiguous genitalia in females
*11β*-hydroxylase deficiency	202010 (8q24.3)	Mutations of *CYP11B1* ^5^	Hyperandrogenism, hypertension
*3β*-hydroxysteroid dehydrogenase type 2 deficiency	201810 (1p12)	Mutations of *HSD3B2* ^6^	Ambiguous genitalia in boys, postnatal virilisation in girls
*17α*-hydroxylase deficiency	202110 (10q24.32)	Mutations of *CYP17A1* ^*7*^	Pubertal delay in both sexes, primary amenorrhea, lack of secondary sexual characteristics, hypertension
P450 oxidoreductase deficiency	201750 (7q11.23)	Mutations of *POR* ^8^	Skeletal malformations, especially craniofacial; severe abnormal genitalia
P450 side-chain cleavage deficiency	613743 (15q24.1)	Mutations of *CYP11A1* ^9^	XY sex reversal
Congenital lipoid adrenal hyperplasia	201710 (8p11.23)	Mutations of *STAR* ^10^	XY sex reversal
Smith-Lemli-Opitz syndrome	270400 (11q13.4)	Mutations of *DHCR7* ^11^	Mental retardation, craniofacial malformations, growth failure, cholesterol deficiency
Adrenal hypoplasia congenita			
X-linked	300200 (Xp21.2)	Mutations of *NR0B1* ^12^	Hypogonadotropic hypogonadism in boys (occasionally in carrier females for skewed X-chromosome inactivation)
Xp21 deletion syndrome	300679 (Xp21)	Deletion of *GK* ^13^, *DMD* ^14^, and *NR0B1*	Duchenne muscular dystrophy, glycerol kinase deficiency psychomotor retardation
*SF1*-linked	612965 (9q33.3)	Mutations of *NR5A1* ^15^	XY sex reversal
IMAGe syndrome	614732 (11p15.4)	Mutations of *CDKN1C* ^16^	Intrauterine growth retardation, metaphyseal dysplasia, genital abnormalities
Kearns-Sayre syndrome		Deletions of mitochondrial DNA	Deafness; heart, ocular and cerebral involvement; skeletal muscle myopathy; intestinal disorders; hormonal deficits
Wolman disease	278000 (10q23.31)	Mutations of *LIPA* ^17^	Bilateral adrenal calcification, hepatosplenomegaly
Sitosterolaemia		Mutations of *ABCG5* ^*18*^ and *ABCG8* ^19^	Xanthomata, arthritis, premature coronary artery disease, short stature, gonadal failure
Familial glucocorticoid deficiency or corticotropin insensitivity syndromes			
Type 1	202200 (18p11.21)	Mutations of *MC2R* ^20^	Hyperpigmentation, tall stature, typical facial features, lethargy and muscle weakness with normal blood pressure
Type 2	607398 (21q22.11)	Mutations of *MRAP* ^21^	Hyperpigmentation, normal height, hypoglycaemia, lethargy, and muscle weakness with normal blood pressure
Variant of familial glucocorticoid deficiency	609981 (8q11.21)	Mutations of *MCM4* ^22^	Growth failure, increased chromosomal breakage, natural killer cell deficiency
Primary generalised glucocorticoid resistance	(5q31.3)	Mutations of *GCCR* ^23^	Fatigue, hypoglycaemia, hypertension, hyperandrogenism
Triple A syndrome (Allgrove’s syndrome)	231550 (12q13.13)	Mutations of *AAAS* ^*24*^	Achalasia, alacrima, deafness, mental retardation, hyperkeratosis
**2) Acquired diseases**			
Bilateral adrenal haemorrhage	-	Meningococcal sepsis, antiphospholipid syndrome	Symptoms and signs of underlying disease
Bilateral adrenal metastases	-	Lung, stomach, breast, and colon cancers	Disease-associated clinical manifestations
Bilateral adrenalectomy	-	Adrenal masses, phaeochromocytoma unresolved Cushing’s syndrome	Symptoms and signs of underlying disease
Bilateral adrenal infiltration	-	Adrenal lymphoma, amyloidosis, haemochromatosis	Disease-associated clinical manifestations
Drug-induced adrenal insufficiency	-	Anticoagulants, ketoconazole, fluconazole, etomidate, phenobarbital, phenytoin, rifampicin, troglitazone	None, unless related to drug
Infectious adrenalitis	-	Tuberculosis, HIV-1, histoplasmosis, cryptococcosis, coccidioidomycosis, syphilis, trypanosomiasis	Disease-associated manifestations in other organs
Autoimmune adrenalitis	-		
Isolated			None
APS type 1 (APECED)	240300 (21q22.3)	Mutations of *AIRE* ^25^	Chronic mucocutaneous candidosis, hypoparathyroidism, other autoimmune diseases
APS type 2	269200		Thyroid autoimmune disease, type 1 diabetes, other autoimmune diseases
APS type 4			Autoimmune gastritis, vitiligo, coeliac disease, alopecia, excluding thyroid disease and type 1 diabetes

Modified by Charmandari et al., 2014 [16]: ^1^OMIM: Online Mendelian Inheritance in Man database; ^2^ *ABCD1*: *atp-binding cassette subfamily D, member 1*; ^3^ *ABCD2*: *atp-binding cassette, subfamily D, member 2*; ^4^ *CYP21A2*: *cytochrome P450, family 21, subfamily A, polypeptide 2*; ^5^ *CYP11B1*: *cytochrome P450, subfamily XIB, polypeptide 1*; ^6^ *HSD3B2*: *3-beta-hydroxysteroid dehydrogenase 2*; ^7^ *CYP17A1*: *cytochrome P450, family 17, subfamily A, polypeptide 1*; ^8^ *POR*: *cytochrome P450 oxidoreductase*; ^9^ *CYP11A1*: *cytochrome P450, subfamily XIA, polypeptide 1*; ^10^ *STAR*: *steroidogenic acute regulatory protein*; ^11^ *DHCR7*: *7-dehydrocholesterol reductase*; ^12^ *NR0B1*: *nuclear receptor subfamily 0, group B, member 1*; ^13^ *GK*: *glycerol kinase*; ^14^ *DMD*: *dystrophin*; ^15^ *NR5A1*: *nuclear receptor subfamily 5, group A, member 1;* ^16^ *CDKN1C*: *cyclin-dependent kinase inhibitor 1C*; ^17^ *LIPA*: *lipase A, lysosomal acid*; ^20^ *MC2R*: *melanocortin 2 receptor*; ^21^ *MRAP*: *melanocortin 2 receptor accessory protein*; ^22^ *MCM4*: *minichromosome maintenance, Saccharomyces Cerevisiae, homolog of, 4;* ^23^ *GCCR*: *glucocorticoid receptor*; ^24^ *AAAS*: *AAAS* GENE; ^25^ *AIRE*: *autoimmune regulator.*

Replacement therapy with standard doses of glucocorticoid (hydrocortisone, 15 mg/m^2^/day), mineralocorticoid (fluorocortisone, 0.2 mg/day), and sodium chloride (NaCl, 1 g/day) was initiated.

Routine cytogenetic investigations revealed an apparently normal female karyotype (46, XX). Molecular karyotyping was performed using an array comparative genomic hybridization analysis using proband's DNA and a 44 K array platform (Agilent Technologies) with a resolution of approximately 100 kilobase. This examination yielded normal results.

**Figure 2:**
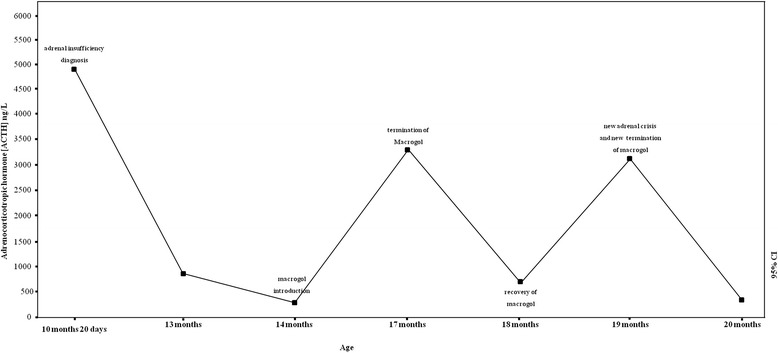
**Corticotropin (ACTH) serum levels (ng/L) at diagnosis of adrenal insufficiency and the start of hydrocortisone (10 months, 20 days of age), after the start of macrogol (14 and 18 months of age), and after the termination of macrogol (17 and 19 months of age).**

After replacement therapy, electrolyte abnormalities were corrected during the first week, and the patient was discharged in good clinical condition. During follow-up, she maintained good condition, good appetite, weight gain, and normal laboratory results with reduced ACTH (Figure 2). After 10 days, ACTH was 3214 ng/L; renin activity, 165.3 μU/mL; Na, 139 mEq/L; K, 4.4 mEq/L; and Cl, 96 mEq/L, and after 40 days ACTH was 797 ng/L.

At 14 months of age, the patient started taking macrogol twice daily for constipation, about 30 to 60 minutes after taking hydrocortisone and fluorocortisone. Testing revealed that ACTH was 300 ng/L, and renin activity was 24.2 μU/mL. Analysis of the *SF1* gene was normal with the exception of a c.437G > C polymorphism.

After 3 months of macrogol therapy, Na was 135 mEq/L; Cl, 106 mEq/L; K, 5.3 mEq/L; renin activity, 124.2 μU/mL; aldosterone, 0.13 nmol/L; cortisol, 1.78 μg/dL; and ACTH, 3262 ng/L. The macrogol was stopped, resulting in a rapid reduction of corticotropin; after 28 days it was 648 ng/L. At this time, faecal elastase was 548 μg/g (normal, > 200 μg/g), and steatocrit was 0% (normal, < 3%). Screening for celiac disease was negative (IgA, 38 mg/dL; tTG, 1.0 U/mL).

Unfortunately, the family arbitrarily reintroduced macrogol (once daily, more than 2 hours after taking hydrocortisone and fluorocortisone) for chronic constipation. After 1 month, during a respiratory tract infection, the child presented with hypoglycaemia, lethargy, weakness, and hypotonia. Vitals were measured: pulse, 134/min; blood pressure, 65/42 mm Hg; and respiration, 38/min. Glucose was 36 mg/dL; Na, 132 nEq/L; Cl, 92 mEq/L; K, 5.4 mEq/L; and ACTH, 3145 ng/L. During recovery, we treated the adrenal deficiency and stopped the macrogol with near normalization of corticotropin (323 ng/L) after 23 days (Figure 2). Neuro-metabolic tests (plasma aminoacidogram, urine aminoacidogram, acylcarnitine profile analysis, and redox state) were again normal.

## Discussion

A variety of laxatives are available for treating constipation: bulk forming, osmotic, and stimulant laxatives. Osmotic laxatives, particularly PEG preparations, are popular because they are relatively safe, inexpensive, and better than lactulose in improving stool frequency and consistency [12-14]. Hydrocortisone is a hydrophilic drug used to treat many conditions, such as primary or secondary adrenal insufficiency, hypopituitarism, and adrenogenital syndrome.

Nevertheless, treatment of children suffering from adrenal insufficiency is frequently problematic for a number of reasons. For example, it requires use of pharmaceutical formulations that do not fully address the pharmacokinetic and pharmacodynamic problems of dosing infants. Therefore, children require careful monitoring of dose and dosage regimen. In fact, patients with adrenal insufficiency continue to have increased mortality and morbidity despite treatment and monitoring [15]. However, many drugs, for example, anticonvulsants such as phenytoin, phenobarbital, and carbamazepine, stimulate cytochrome P450 3A4, induce hepatic enzymes, and lead to accelerated glucocorticoid metabolism and reduced glucocorticoid effect, possibly causing acute adrenal insufficiency [16].

Hydrocortisone preparations are commonly combined with pharmaceutically acceptable carriers, typically inert, to facilitate their administration. Polyethylene glycol contains a mixture of inert water-soluble molecules of different sizes, whose absorption is independent of dosage, displaying decreasing mucosal transport with increasing molecular size. Macrogol solutions are commonly used for their efficacy and low rate of absorption (0.2%) after oral administration [17] and typically have a safe profile with minimal reported side effects.

A drug’s solubility in water is an important factor influencing its release into the body. In addition, macrogol softens the faecal mass by osmotically drawing water into the GI tract. As our case showed, it is possible that macrogol reduces the absorption of hydrocortisone, facilitating the appearance of adrenal insufficiency. The case seems to support our hypothesis, considering the significant changes in corticotropin after starting and stopping macrogol. Furthermore, we could also speculate that the introduction of macrogol close to that of hydrocortisone or fluorocortisone could cause or contribute to the reduced absorption of these drugs, triggering the adrenal crisis. In fact, it is recognised that many physiological gastrointestinal factors may strongly influence the plasma concentration-time profile of hydrocortisone [18]. However, hydrocortisone has a high permeability in both the small and large intestines, and the short elimination half-life (near 1.5 h) requires two or more dose administrations per day [18].

This aspect is of great concern because patients with primary or secondary adrenal insufficiency have more than twofold increased mortality than the general population. However, recent data have demonstrated that the metabolic cardiovascular risk in hypopituitarism is related to the daily dose of hydrocortisone [15].

Our case report, while not demonstrating a genetic aetiology (polymorphism of *SF1* was the only abnormality), gives evidence of a possible genetic primary cause of adrenal insufficiency, based on clinical and laboratory examinations and the age of onset. In children, congenital primary adrenal insufficiency is very rare, accounting for about 1% of all cases. The importance of elucidating a genetic basis is emphasised by the ever-increasing number of genetic causes of adrenal insufficiency (Table 1) [16]. In fact, in a series of 103 children with Addison’s disease, genetic forms were very frequent, accounting for 72% of congenital adrenal hyperplasia; other genetic causes accounted for 6%, whereas autoimmune disease was diagnosed in only 13% [19].

As stressed by this case, prompt diagnosis is also important because acute adrenal insufficiency is a life threatening disease. Typically, patients with this disease present with severe hypotension to hypovolaemic shock, vomiting, acute abdominal pain, and often fever. However, children often present with hypoglycaemia and hypoglycaemic seizures. On the other hand, the primary non-specific symptoms of chronic adrenal insufficiency in children are fatigue, reduced muscle strength, weight loss, anorexia, or failure to thrive [20].

## Conclusions

This case report suggests that macrogol 3350 could interfere with the absorption of hydrocortisone. It is of particular importance considering the risk of adrenal insufficiency in these patients, and careful attention should be paid to the concomitant use of macrogol and hydrocortisone in subjects with primary or secondary glucocorticoid deficiencies.

## Consent

Written informed consent was obtained from the parents of the patient for publication of this Case Report and any accompanying images.
